# Mechanistic Aspects and Effects of Selected Tank-Mix Partners on Herbicidal Activity of a Novel Fatty Acid Ester

**DOI:** 10.3390/plants11030279

**Published:** 2022-01-21

**Authors:** Javier Campos, Luciana Bodelon, Mercedes Verdeguer, Peter Baur

**Affiliations:** 1 Global Innovation & Technology, Clariant, 65926 Frankfurt am Main, Germany; Luciana.Bodelon@clariant.com; 2 Instituto Agroforestal Mediterráneo, Universitat Politècnica de València, 46022 Valencia, Spain; merversa@eaf.upv.es; 3 CropPromotion, 86938 Schondorf am Ammersee, Germany; profpeterbaur@icloud.com

**Keywords:** contact herbicide, pelargonic acid, esterified seed oil, foliar penetration, adjuvant, tank-mix partner

## Abstract

Only a limited number of contact herbicides exist in agricultural production. While systemic herbicides are more efficient also at suboptimum spray coverage with long-lasting weed control, contact herbicides provide several advantages. There is no translocation to fruits or roots of plantation and other crop, low risk for resistance development, and minor risk for spray-drift damage. Besides, synthetic products that often have toxicological or residues issues, natural fatty acids, particularly pelargonic acid (PA), have contact activity and are safer for home and garden use. We recently described a methyl capped polyethylene glycol ester of pelargonic acid (PA-MPEG) that acts independent of acid formation. Both, PA-MPEG and PA are applied at high rates per hectare to achieve excellent weed control. Here, we report about potential additives to increase PA-MPEG efficacy. The herbicidal active, *1*-decanol, and the non-phytotoxic alkylated seed oil-based adjuvant, Hasten^TM^, improved performance and outperformed a commercial PA herbicide. Both, PA-MPEG and PA appear to mainly act by the disintegration of bio-membranes besides having effects on transpiration. The main suggested effect is desiccation due to cutting the water continuum at the site of evaporation in the intercellular spaces. The synergistic action of the adjuvant Hasten^TM^ and its practical uses are also discussed.

## 1. Introduction

Most herbicides normally used for agricultural weed control are based on synthetic active ingredients (AIs) and possess systemic properties [1,2]. The majority of foliar AIs are low to moderately water-soluble non-electrolytes, with an octanol/water partition coefficient (log *P*) below 4, that allows acropetal movement in the xylem. Other active substances are weak organic acids or form such acids from pre-drug esters that move both basipetally and acropetally through the plant (mobility in the phloem) [2]. In many cases, systemic soil-applied herbicides are only xylem mobile after root or hypocotyl uptake, and/or when sufficiently volatile to also distribute in the gas phase of soils [2,3]. Selective weed control by such systemic herbicides is based on several complex and sophisticated plant-herbicide interactions, such as herbicide-tolerant transgenic crops, combinations with safeners, timing of applications or tolerance by a higher biomass, or the quick growth compensation of damaged assimilation areas [1,2,4,5,6].

In contrast to more effective and systemic AIs, only a few available herbicides are not translocated in plants. They are commonly known as contact herbicides and act only on treated organs [1,6]. The most important ones are quaternary ammonium compounds of bipyridines, and 1,1’-dimethyl-4,4’-bipyridylium dichloride (paraquat) is the most widely used [7]. The advantages of such compounds are the rare occurrence of herbicide-resistant weeds (paraquat resistance is documented in 30 plant species in 72 situations vs. 53 species in 339 scenarios for glyphosate use) and the low risk that sensitive weeds become resistant over generation [8]. Other benefits are that they are harmless to non-target plants by spray drift and their lack of translocation, e.g., to fruits in orchards [9]. However, given the bipyridines’ mode of action (MoA)—formation of reactive oxygen species after accepting electrons from photosystem I, causing the inhibition of photosynthesis—there are concerns regarding safety. Nonselective, nonspecific damage and continuous action occur due to the active ingredient regeneration causing oxidation of cell components, including membranes. The toxicity and side effects of these herbicides on human and non-target organisms are substantial [7]. Therefore, paraquat and related products are increasingly banned in various regions [7,10].

Contact herbicides also include natural alternatives with practically zero toxicity such as nonanoic or pelargonic acid (PA) and related octanoic (caprylic) and decanoic (capric) acids [11,12,13]. Several commercial formulations of short chain fatty acids (FA) and their salts are available for weed control [14,15,16]. With the advantage of having extremely rapid action and being rainfast, they do not pose residual problems, and no resistant weed biotypes have been reported [17,18,19]. However, FA herbicides are volatile, have an unpleasant odor and are difficult to formulate [20,21]. For good and long-lasting effects on weed control, FA should be applied at extremely high rates, and repeated applications must be performed within short time intervals, which makes them very expensive for users [22,23,24]. Given their fast herbicidal activity, the combination with other synthetic or natural AIs is a challenge. It is often impossible to achieve a synergistic or an additive effect on weed control, particularly with systemic AIs [25,26].

We have recently shown that novel short chain FA derivatives, particularly the methyl polyethylene glycol esters (MPEG) of C_8_–C_10_ FA, are as effective as the free acid, and do not merely act as pre-drug [23]. Pelargonic acid ester of methyl polyethylene glycol (PA-MPEG) is the preferred candidate [23]. PA-MPEG is liquid at the relevant temperature range, not volatile and can be used as a straight product without further formulation efforts [23]. It is also combinable in-can or in tank-mix with other herbicides and acts as a wetting agent on its own [27]. With its very low animal and human toxicity as known to date, the use is very encouraging in environmentally friendly and organic farming. The use rate and water volumes of PA-MPEG are lower than those of various current PA formulations, but are still higher than conventional herbicides [17,23]. Therefore, further reductions in the use rate and spray volume, and increasing PA-MPEG performance, are essential for it to become an alternative to the traditional contact herbicides.

Adjuvants and natural additives are often added to the spray tank of the herbicides to enhance final performance. They can modify the characteristics of the spray mixture or improve herbicidal activity [28,29]. Thus, adjuvants also have the potential to enhance PA-MPEG activity by affecting spray deposition, bioavailability and/or the effect in the transport across cuticles [28,29,30,31]. For example, a strong selected wetting agent can offer better coverage, which is fundamental for contact herbicides, or an alcohol ethoxylated can increase the mobility of a solute in cuticles [29,30].

In this study, we present the results of sustainable adjuvants and natural additives as potential enhancers of PA-MPEG weed control efficacy. New insights into the likely mode of action are also discussed.

## 2. Materials and Methods

### 2.1. Plant Species and Biological Test Conditions

Seeds of velvetleaf (*Abutilon theophrasti* M.), large crabgrass (*Digitaria sanguinalis* L.) and black nightshade (*Solanum nigrum* L.) were acquired from Herbiseed (Reading, UK). Tomato (*Solanum lycopersicum* L.), bell peppers (*Capsicum annuum* L.), soybean (*Glycine max* L.) and maize (*Zea mays* L.) seeds were kindly provided by a local farmer in Bad Soden am Taunus (Germany). Plant species seeds were sown separately in plastic pots (9 cm × 9 cm × 10 cm) containing an artificial substrate named Typ B Hawita Fruhstorfer from Hawita Gruppe GmbH (Vechta, Germany). One week after emergence, plants were carefully thinned to one plant per pot. Weeds and crops were grown in the Clariant phytotron (Frankfurt am Main, Germany) under natural light and augmented with supplemental sodium vapor lights that produced a photosynthetic photon flux density (PPFD) of 200 mE m^−^^2^ s^−1^. The photoperiod was 16/8 h light/dark. Daytime temperature was 23 ± 1 °C, and night-time temperature was kept at 18 ± 1 °C. Relative humidity (R.H.) fell within 55 ± 5% range. Enough moisture was maintained in soil until the end of trials to avoid water stress and keep plants in the optimum stage. Crop plants were irrigated with a standard fertilizer solution once a week to prevent nutrient deficiencies.

### 2.2. Experimental Design of the Phytotron Trials

Trials were conducted as a randomized complete block (RCB) design with four replicates per weed species. An untreated control was always included for comparison purposes. Spray preparations were applied to *D. sanguinalis* in phenological stage 22 (with two tillers) and *S. nigrum* in stage 16 (true six leaves) according to the *Biologische Bundesanstalt, Bundessortenamt und Chemische Industrie* (BBCH) scale. They were approximately 18–20 cm tall. Applications were carried out with a custom-built spray chamber (Ing-Büro CheckTec, Braunschweig, Germany) equipped with two off-center flat nozzles and a mobile carrier of the spray tank. Spray volumes of 200 and 400 L ha^−1^ were set by adapting the application carrier speed, using OC2 nozzles from Lechler GmbH (Metzingen, Germany) mounted 50 cm above the weed canopy. Spray pressure was 300 kPa.

Herbicide efficacy was visually assessed at 1, 2 and 7 days after application (DAA) on a percentage scale, where the value “0%” represents no weed control (weeds alive) and one of “100%” denotes complete weed control (weeds killed) [23].

#### 2.2.1. Herbicidal Compound and Tank-Mix Partners Tested

The experimental herbicide was the pelargonic acid ester of methylated polyethylene glycol (PA-MPEG) which was synthesized by Clariant (Gendorf, Germany). This active is liquid and was diluted directly in tap water. For comparison purposes, PA-MPEG content is 340 g of PA acid equivalent (a.e.) per liter. Based on previous knowledge, PA-MPEG was used at 7.5% *v/v*, alone or with selected tank-mix partners, at a spray volume of 200 L ha^−1^ [17]. Phosphoric acid, D-glucose, potassium carbonate and *1*-decanol were selected as non-synthetic amendments. They were purchased from Sigma-Aldrich Chemie GmbH (Merk KGaA, Darmstadt, Germany). The tested commercial adjuvants were Synergen^®^ TS 7, Polyglykol 400, Genapol^®^ C 050 from Clariant ((Muttenz, Switzerland) and Hasten^TM^ from Victorian Chemicals (Victoria, Australia) [30,31]. Table 1 is a more detailed description of the tested compounds and the applied rates.

#### 2.2.2. Interaction of the Hasten Concentration and the PA-MPEG Rate and Spray Volume

Two experiments were carried out to check the optimum conditions for the PA-MPEG and Hasten. The first trial was conducted with a factorial arrangement of three Hasten use concentrations (0, 1, 2, and 2.5% *v/v*), two spray volumes (200 and 400 L ha^−1^) and a single PA-MPEG concentration of 7.5%. A commercial emulsifiable concentrate (EC) formulation of PA (Beloukha, 680 g AI L^−1^, Belchim Crop Protection, Londerzeel, Belgium) according to the label recommendation (10.9 kg a.i ha^−1^), was used as a standard reference [14]. In the second experiment, different PA-MPEG concentrations alone or with 2.5% Hasten were applied at 200 L ha^−1^. Based on earlier studies, the following herbicide concentrations were employed: 5, 6, 7, 8, 9, and 10% [17]. No commercial reference was used because we explored Hasten enhancement at different PA-MPEG concentrations and PA-MPEG and the commercial PA herbicide gave closer weed control values at the selected 200 L ha^−1^ in previous trials [17].

#### 2.2.3. Phytotoxicity of Spray Tank Partners after Spraying and Single Droplet Application

The species used for this experiment were *D. sanguinalis* and *S. nigrum,* as described in 2.1. The tested tank-mix partners were mixed in tap-water at the aforementioned concentrations (Table 1). No herbicide (PA-MPEG) was employed in the spray solutions at this time. Test preparations were applied by spraying the weeds and also using 10 μL droplets. In the first experiment, spray applications were performed in the customized spray chamber with the parameters described in Section 2.2. (OC2; 300 kPA; 200 L ha^−1^). Treatments were replicated four times per weed species. The second trial evaluated the phytotoxicity of a single droplet application on the adaxial leaf surface of weeds at room temperature (25 °C and 56% RH). In addition, 10 μL droplets were also applied on the adaxial leaf of *A. theophrasti* (*BBCH 14*), tomato (BBCH 16), soybean (BBCH 16), and maize (BBCH 14), whose characteristics and wettability differ. An adjustable volume pipette (Eppendorf, Hamburg, Germany) was used for droplet applications. Two leaves were treated per plant, and two plants were treated for each plant species (four leaves in tall for each plant species). After droplet evaporation, plants were placed in the phytotron. Phytotoxicity was visually evaluated 1 day after treatment and was then assessed as described in Table 2.

### 2.3. Laboratory Experiments

#### 2.3.1. Cuticular Penetration

The penetration tests of PA-MPEG and free PA (99%, Matrica, Porto Torres, Italy), with and without additive, were studied with enzymatically isolated cuticular membranes as described in detail in the literature [32,33]. Mature leaves of apple trees (*Malus domestica* B.) cv. Gala, from plantations in Hofheim am Taunus (Germany), were taken in June, and after a quick transfer to the laboratory, 2-cm diameter discs were punched with cork borers. Leaf discs were vacuum-infiltrated in a pectinase-cellulase solution. After incubation in the enzymatic solution for about 2 weeks, cuticles were separated, cleaned with deionized water, and dried on Teflon plates.

Adaxial cuticles (stomata-free) were mounted on stainless steel chambers with original outer surfaces exposed to air, and the inner cuticle surface came into contact with the aqueous-acceptor solution from the chamber’s interior [32]. Under controlled conditions (25 °C and 56% R.H.), 10 μL droplets of the spray solution were applied to the external cuticle surface of the cuticles and dried in room ambient with air circulation (approx. 25 min.). The aliquots of the acceptor solution that were drawn after different time points were analyzed by a 1290 Infinity HPLC (Agilent, Santa Clara, CA, USA).

A geometric mean of the penetration values per treatment was obtained from 10 repetitions and three measurements (6, 24, 48 h after application). The kinetics indicated the mean of active ingredient penetration across the cuticle at different times.

#### 2.3.2. Characterization of Spray Deposits on Glass Slides

Spray deposits of PA-MPEG and PA with and without inert ingredients were characterized on silanized glass slides on parallel to the cuticular penetration test. The physical appearance of the 10 μL droplet was analyzed with a research light microscope (DM4000M, Leica, Wetzlar, Germany) in the polarized light modus connected to a high-resolution color digital camera (DFC450, Leica).

#### 2.3.3. Scanning Electron Microscope (SEM)

The adaxial leaf samples of *D. sanguinalis* and *S. nigrum* were observed by a scanning electron microscope JSM-5600 LV from JEOL (Tokyo, Japan). Test preparations (0.3 μL droplets) were applied to leaves. After allowing for water evaporation under room conditions (25 °C and 56% R.H.), for approx. 30 min., samples were prepared as described in detail by Pathan et al. 2010 [34], frozen at −170 °C and sputtered with gold. Then samples were analyzed at different magnifications. The resulting image of the adaxial leaf surface showed minimal distortion, which allowed the product deposit characteristics on leaves to be examined.

#### 2.3.4. Cuticular Transpiration

The effect of PA and PA-MPEG on cuticular transpiration was measured with the enzymatically isolated cuticles of mature ivy (*Hereda helix* L.) leaves. The method first determined transpiration in the steady state before treatment, and then after applying and drying the test compounds. In this experiment, 10 repetitions (individual cuticles) were performed, where each cuticle is control (without treatment) and later treated, allowing paired observations. This method is described in detail in the literature (e.g., Geyer and Schönherr [35].

#### 2.3.5. Stomatal Conductance

The impact of PA-MPEG on leaf transpiration was investigated on bell pepper leaves because they have stomata on both leaf sides, with lower adaxial density, which are similar to *S. nigrum,* but they do not have trichomes (*S. nigrum* leaves have them) that can interfere with porometer measurements. Stomatal conductance was measured adaxially and abaxially with an SC-1 Leaf Porometer (Meter, Pullman, US) at room temperature (25 °C and 56% R.H.). PA-MPEG was only adaxially applied as 10 µL droplet, which was spread over an area of about 1 cm^2^. Porometry measurement was carried out after droplet evaporation on the treated surface (adaxial) and on the abaxial side for the first four hours after application.

### 2.4. Statistical Analysis

The results of the efficacy trials were subjected to an analysis of variance (ANOVA) using the ARM software (Gylling Data Management Inc., Brookings, OR, USA). The individual treatment means were compared by the Student-Newman-Keuls least significant difference (LSD) test at the 5% level of significance (*p* < 0.05). Prior to the analysis, data normality and homoscedasticity were verified using the software’s functionalities. Data were automatically transformed by the software whenever necessary. Data transformations are indicated in the Tables as footnotes.

## 3. Results and Discussion

### 3.1. PA-MPEG Herbicidal Activity Affected by the Test Compounds Added to the Spray Tank

We have previously reported that PA-MPEG is not just a pre-drug of PA, in contrast to the esters of auxins [23]. For example, the *iso*-octyl ester form of 2,4-D (2,4-dichlorophenoxyacetic acid) is rapidly hydrolyzed to free acid, which is the active [36]. Other PA ester derivatives have not shown any herbicidal activity [23]. A comparable extremely rapid action with symptoms of wilt and necrosis on treated organs only a few hours after application, is observed with both PA and PA-MPEG [23].

Various adjuvants have been tested with PA in other studies [37,38]. The impact of salts vs. free acid was also tested [39]. As far as we know, no significant economically reasonable PA efficacy enhancement is known by means of formulation or using tank-mix adjuvants. Since PA-MPEG is different from PA, being potentially both, pre-drug and drug, nonelectrolyte, surface-active, and having a molecular weight 2.5-fold higher [23], we also explored some potential enhancers of its herbicidal activity. Previous works have shown that PA-MPEG efficacy is best at 10% concentration with a 500 L ha^−1^ spray volume on tested weeds [17,23]. We evaluated the weed control of the test compound at the 7.5% concentration with 200 and 400 L ha^−1^ volumes on medium-sized plants (approximately 18–20 cm tall). Table 3 and Table S1 report the effect of the test compounds on weed control 2 days after PA-MPEG application. We also determined weed control after 1 week, but values were not significantly different and gave no further information on the performance of the test compounds.

Except for *1*-decanol and Hasten, the proved products did not enhance the PA-MPEG activity at the selected and tested concentrations. These concentrations were chosen based on economic and potential maximum activity considerations. The extreme spray pH values of pH 2 with phosphoric acid and pH 10 with potassium carbonate (Table 1) did not affect PA-MPEG activity. Previous stability trials suggested that there is no hydrolysis of PA-MPEG in the spray liquid until two days at pH below 10 [37]. Acid hydrolysis did even not occur on the time scale of weeks to months. Spray droplet evaporation was extremely quick and bulk droplet evaporation took only minutes [40]. However, the resulting spray deposit was hydrated because PA-MPEG is a liquid. Alkaline hydrolysis was particularly considered a possible process in the more concentrated spray deposit, resulting in free PA with differentially response. Nevertheless, the obtained results (Table 1) suggested that this was not the case. Thus, potential pH changes in the apoplast by phosphoric acid or potassium carbonate did not affect PA-MPEG stability or efficacy, respectively.

Glucose, as both an osmotic agent and a potential energy source for epiphytic organisms by enhancing the decay of damaged leaves, and PEG 400 as a hygroscopic liquid were neither effective. Since coverage is the key for the herbicidal activity of contact herbicides, another promising candidate was Synergen TS 7, which is a very strong wetting and spreading agent. However, it did not sufficiently improve PA-MPEG efficacy, but obtained better results on *D. sanguinalis,* with a slight increase in weed control than in *S. nigrum,* for which no additional control was observed. No superior spreading probably took place in the presence of the high already PA-MPEG concentration, which is also a wetting agent on its own [23,27]. The lack of any significant influence on PA-MPEG efficacy by strong penetration enhancer, Genapol C 050, was at first surprising [41,42]. However, in the presence of 7.5% PA-MPEG in the spray, and assuming a cuticle/water partition coefficient of Genapol C 050 close to 1, the explanation of these results was that Genapol C 050 probably did not simply enter through leaf cuticles [43,44]. An 7.5% PA-MPEG application at a spray volume of 300 L ha^−1^ by assuming 10% coverage on leaf area, resulted in an AI load of approximately 3 mg cm^−2^. This was 10- to 100-fold more than the cuticle specific mass and was, therefore, more than 10-fold in favor of the spray deposit [45]. Hence, the amount of Genapol C 050 sorbed in cuticles was only 1% of the cuticle mass, or lower. This was too low to increase AI mobility in cuticles, where 5% is needed to obtain significant effects [33,44]. Hasten and *1*-decanol are much more lipophilic, with a log *P* values of 4.5 for *1*-decanol and one above 8 for Hasten. Therefore, both products have a better potential to be quickly sorbed in cuticles after spray applications.

### 3.2. Phytotoxicity of 1-Decanol and Hasten

These two test compounds were also examined for their own phytotoxicity after spraying on complete plants and also applying individual 10 µL droplets on the leaves of the selected plants. The droplet volume was large, and thus doses per area were much higher than with real sprays. Coarse spray droplets have mean droplet diameters close to 500 µm, and most droplets are typically below the 0.5 µL volume [46,47]. While *1*-decanol is usually applied as a plant growth regulator for the sucker control of tobacco (*Nicotiana tabacum* L.) plants and also can act as a contact herbicide, Hasten is an adjuvant that boosts the efficacy of many AIs without own activity [31,48,49]. This was also reflected by the phytotoxicity results of both these products on the selected weeds and crops, respectively.

**Figure 1:**
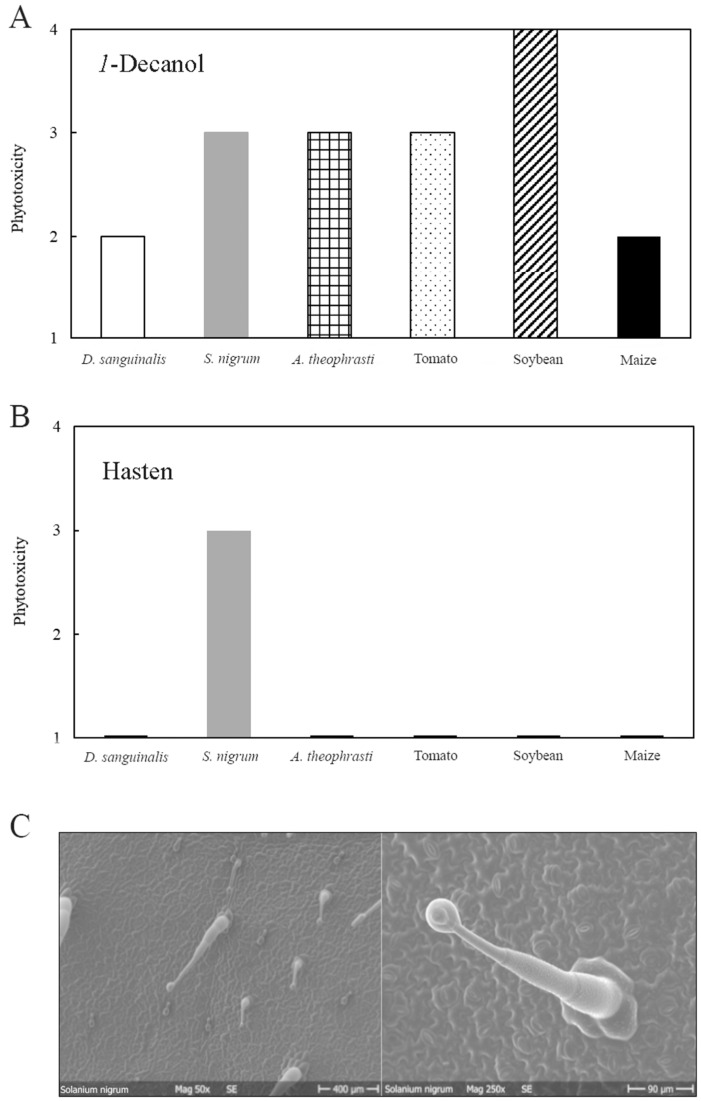
Phytotoxicity of the 10 µL droplet application on the adaxial side of mature leaves after 24 h. (**A**) *1*-Decanol at 1%. (**B**) Hasten at 2.5%. (**C**). SEM micrographs showing the glandular trichomes on the adaxial leaf of *Solanum nigrum*.

On all tested weeds and crops, *1*-decanol at 1% caused phytotoxicity symptoms when applied as an individual droplet (Figure 1A), while spray application did not lead any damage. The typical *1*-decanol use concentration, e.g., for sucker control in tobacco is above 3% [48]. In this experiment, necrotic tissues were already observed at the 1% use concentration due to the 2- to 3-fold higher dose rate per area with 10 µL droplets. *1*-decanol volatility is very high with a vapor pressure of 1387 mPa, while PA-MPEG is non-volatile [23,50]. The log *P* of PA-MPEG is around 2.5, while, *1*-decanol has 100-fold higher lipophilicity and has therefore, completely different bioavailability characteristics. Alcohols with chain lengths of C_8_-C_12_ also increase mobility in the cuticles of other solutes such as PA-MPEG [44,51]. Adding of *1*-decanol to PA-MPEG enhanced its herbicidal efficacy probably by causing additional penetration besides the desiccation effect provoked by *1*-decanol itself.

The situation was completely different with the adjuvant Hasten. This product is very safe according to safety data sheet information and also possesses no herbicidal activity at typical use concentration up to 1% [31,49,52,53]. When sprayed on plants, no phytotoxicity symptoms were observed up to the highest tested concentration (5%), which also indicates no own herbicidal activity. No phytotoxicity symptoms were observed on five of the six selected weeds and crops tested after droplet application of 2.5% Hasten (Figure 1B). Only *S. nigrum* showed necrotic spots after applying 10 µL droplets, while spraying Hasten at even 5% exhibited no symptoms such as leaf curling, yellowing, or necrosis. The *S. nigrum* results were relevant because it is considered a strong allelopathic plant with herbicidal active secondary metabolites [54]. The glandular trichomes (Figure 1C) that exist on both leaf sides contain products such as flavonoids and alkaloids that could be released and enter leaf tissue to cause phytotoxicity even on *S. nigrum* itself [54]. Therefore, it is likely that the presence of Hasten caused the release of the allelopathic herbicidal compounds from *S. nigrum* trichomes, and it enabled to enter the mesophyll tissue of the leaf. When trichomes were damaged by a razor blade, no symptoms such as necrosis were developed in the absence of Hasten, but symptoms appeared when 2.5% Hasten was later applied. Apparently, Hasten also acted as a penetration enhancer for substances in glandular trichomes, but it did not cause phytotoxicity on its own [53].

### 3.3. Concentration Dependence of the Adjuvant Effect on PA-MPEG Herbicidal Activity

**Figure 2:**
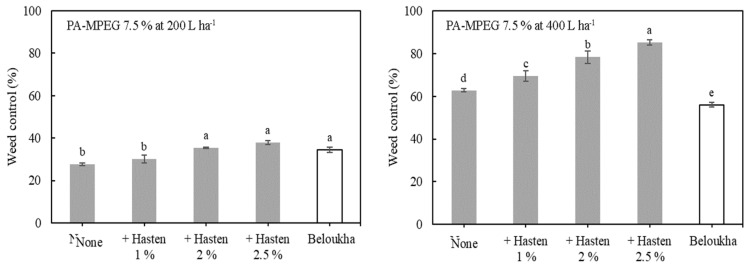
Effect of the Hasten rate and spray volume on the weed control of *Digitaria sanquinalis* with pelargonic acid ester of methylated polyethylene glycol (PA-MPEG) at 7.5%. Visual assessment at 2 days after application. Common letters above bars indicate that the means are not significantly different by the Student-Newman-Keuls test at the 5% level. Bars represent standard errors. Beloukha’s rate was 10.9 kg a.i. ha^−1^.

After considering the positive effect of Hasten on the herbicidal activity of PA-MPEG, this adjuvant was used in further experiments. As previously mentioned, PA-MPEG was tested at 7.5% instead of at the previously reported optimum 10% PA-MPEG to better differentiate its herbicidal activity [23]. The employed benchmark, Beloukha, was a high load PA formulation (680 g/L) applied at the recommended use rate and water volumes (Figure 2 and Table S2). With a 400 L ha^−1^ spray volume, Beloukha (10.9 kg a.i ha^−1^) showed an inferior efficacy than PA-MPEG at 7.5% (10.2 kg a.e ha^−1^). However, at 200 L ha^−1^ both PA-MPEG 7.5% (5.1 kg a.e ha^−1^) and Beloukha achieved very low level weed control. The addition of Hasten at 1.0–2.5% to the spray tank positively affected the PA-MPEG efficacy in a concentration-dependent way. Weed control increased for both the water volumes tested up to 20%, with slightly stronger effects on weed control percentage at 400 L ha^−1^. With Hasten, PA-MPEG performance was clearly boosted and superior to the commercial PA formulation (Figure 2 and Table S2). Obviously, the effect of water volumes dominated the differences in PA vs. PA-MPEG, and the adjuvant’s impact on PA-MPEG efficacy [17]. On the other hand, PA-MPEG efficacy was increased by the adjuvant even at 400 L ha^−1^, reaching a higher weed control level after 2 days, and being approximately 30% better than the benchmark.

The beneficial impact of the larger water volume was not related to coverage *per se*, i.e., the absolute area of the treated weed plant surfaces. This was practically complete at 200 L ha^−1^ for *D. sanguinalis* after treatment with both products, PA-MPEG and the benchmark. Both, spray liquid adhesion and capillary wetting of monocots with surfactant solutions below critical surface tension (35 mM m^−1^) ensure full treated leaf area coverage [55]. Spraying with fluorescent tracers displayed full coverage [17]. At a higher load liquid (400 L ha^−1^) there was more run-off to the leaf angles of the vertical grass leaves [39]. So, the better performance of 7.5% PA-MPEG at the 400 L ha^−1^ spray volume could be caused by the higher dose per area of PA-MPEG and its basipetal run-off of spray liquid with uneven distribution [17,55].

**Figure 3:**
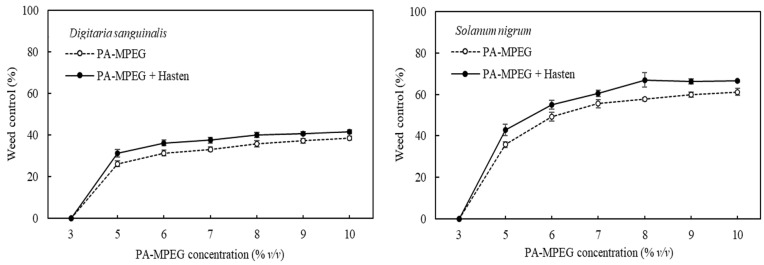
Effect of 2.5% Hasten on the weed control of *Digitaria sanguinalis* and *Solanum nigrum* at different concentrations of pelargonic acid ester of methylated polyethylene glycol (PA-MPEG), 2 days after application. Spray volume of 200 L ha^−1^. Bars represent standard errors.

At the 2.5% adjuvant concentration and the 200 L ha^−1^ spray volume, we examined the optimum use concentrations of PA-MPEG on *D. sanguinalis* and *S. nigrum* (Figure 3 and Table S3). Previous results have not shown either herbicidal activity or phytotoxicity at 3% PA-MPEG [17]. While the maximum control with 10% PA-MPEG was not exceeded much by adding Hasten at 2.5%, there was a consistent increase at lower use PA-MPEG concentration. The results suggest that 7.5% PA-MPEG plus the adjuvant was comparable to 10% PA-MPEG. The enhancing effect of Hasten was given at all PA-MPEG concentrations for both weeds, but there was no hint for a particular ratio for optimum increases.

### 3.4. Pelargonic Acid and PA-MPEG Cuticular Penetration

**Figure 4:**
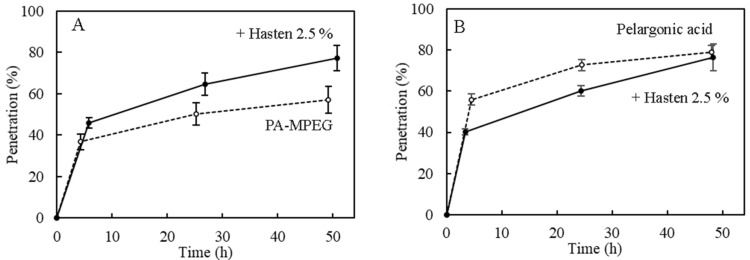
The impact of Hasten at 2.5% on the cuticular penetration of (**A**) pelargonic acid ester of methylated polyethylene glycol (PA-MPEG) at 25 g a.e. L^−1^ and (**B**) straight pelargonic acid (PA) at 25 g a.i. L^−1^. Each curve is the mean of seven to nine repetitions. (Temperature was 25 °C and relative humidity was 56%). Bars represent standard errors.

Previous studies have demonstrated that Hasten does not significantly enhance PA activity [37]. We have also found that it is neutral and sometimes antagonistic for salts such as ammonium PA and C_8_–C_10_ FA at equal amounts of active substance per hectare [39]. In contrast, PA-MPEG performance was significantly improved by Hasten (Figure 2 and Figure 3 and Table S3). As wetting agent related effects and others such as drift or volatility can be excluded, we checked the potential effects on PA-MPEG penetration compared to free PA. Figure 4 illustrates how Hasten acts as penetration enhancer of PA-MPEG but conversely decreased PA penetration, which was faster penetrating than PA, with about 30 % more absorbed a few hours after application. The penetration level, at the very high doses per area, corresponding to the 25 g a.e. L^−1^ solute concentration, was also generally high. The difference in penetration correlated well with the observed shifts in herbicidal activity in the presence of the adjuvant. Hasten belongs to the class of alkylated or methylated seed oil (MSO) type adjuvants that are swelling agents for cuticles [32]. This increases the mobility of the AI and a several-fold faster penetration through more liquid-like cuticles [44].

In the presence of Hasten, PA-MPEG reached the PA penetration level after 2 days, while it was still slightly below PA at shorter times. The penetration of both PA and PA-MPEG was very fast and similar to the quickly penetrating alcohol ethoxylates, such as the previously mentioned Genapol C 050, where a fraction of 60–80% of the applied amount penetrates within one day the cuticle of different species [51]. Free PA is a very small molecule with 110 cm^3^ mol^−1^ [20]. The PA mobility is so high that adjuvants such as Hasten cannot increase mobility [44]. The negative effect of Hasten on PA penetration was similar to the one observed effect with Genapol C 050 on PA-MPEG, and it is probably related to a change of partitioning coefficient [32,44]. The mixtures of Hasten with large amounts of PA reduced the sorption in cuticles. In contrast to the free PA, PA-MPEG is a bulkier molecule with a molecular weight over 400 g mol^−1^ which results in a 3-fold bigger molar volume than PA. Although linear molecules were found to have higher mobilities, this does not apply to PA-MPEG, which has a central ester group and, thus, sp2 hybridization. It was found that ethoxylates of fatty acids (esters) having the same degree of ethoxylation penetrate much slower than alcohol ethoxylates (ethers). This structure and molecular weight caused a 10-fold lower mobility in cuticles. Clearly, the swelling effect of Hasten increased mobility in such a way that enhanced cuticular permeability resulted in better weed control [44].

### 3.5. Characterization of Spray Deposits on Glass Slides

**Figure 5:**
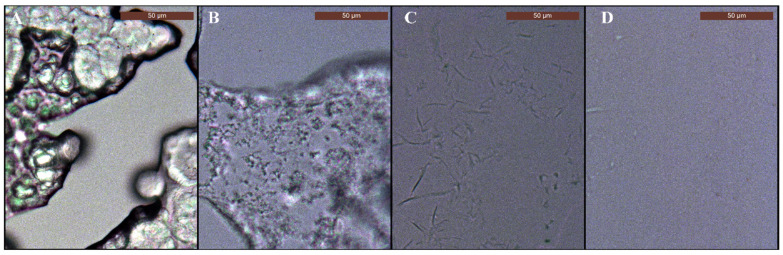
Optical microscope images of spray deposits on glass slides. (**A**) Straight pelargonic acid (PA) at 25 g a.i. L^−1^, (**B**) PA plus Hasten 2.5%, (**C**) pelargonic acid ester of methyl polyethylene glycol (PA-MPEG) at 25 g a.e. L^−1^, and (**D**) PA-MPEG plus Hasten 2.5%.

Spray deposits showed a homogeneous and amorphous PA-MPEG and Hasten mixture, which indicates good bioavailability (Figure 5). The light microscopic evaluation of deposits on glass slides suggested that PA always forms some crystalline particles with counterions from water, which were also visible in the presence of Hasten (Figure 5).

### 3.6. Scanning Electron Microscope (SEM)

**Figure 6:**
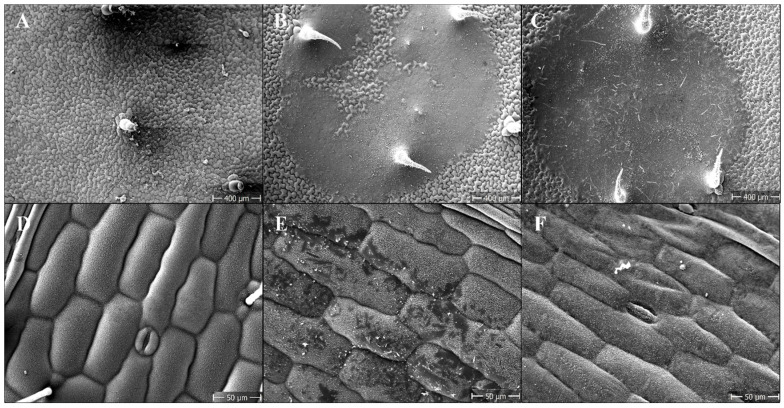
SEM micrographs of the *Solanum nigrum* (upper row) and *Digitaria sanguinalis* (lower row) leaves, 2 h after the 0.3 μL droplet application of pelargonic acid ester of methyl polyethylene glycol (PA-MPEG) at 25 g a.e L^−1^ with (**C**,**F**) and without (**B**,**E**) Hasten 2.5%. Untreated leaves (**A**,**D**).

Figure 6 shows for single droplet application that Hasten closed the gaps not covered with PA-MPEG in the spray droplet deposits on *S. nigrum* leaves, which could result in the recovery of the leaf tissue below. On *D. sanguinalis*, the deposit area also appeared more homogeneous than with the straight PA-MPEG application.

### 3.7. The Mechanistic Aspects and High Use Rates of PA-MPEG and PA

The high use rates of both PA-MPEG and PA are still a limiting factor for their use in conventional crop production, even though the products are comparable in costs per hectare to for example, glufosinate, and are more environmentally friendly. PA is quite volatile, and loss of product could be one reason for the necessity of high PA rates. However, also for non-volatile PA-MPEG, high rates are needed for good weed control [17]. So, this property does not appear to be very relevant. The phytotoxic symptoms with both PA and PA-MPEG typically start with wilting several hours after application and desiccation of the treated plant parts and, if sufficiently extensive, weed death. A second application is sometimes needed to exhaust weeds. The generally suggested MoA for PA and PA-MPEG are changes in the leaf epidermal structure, such as erosion of surface waxes, a related higher leaf transpiration, the disintegration of bio-membranes, and likely as a consequence, decreased photosynthesis [13,37].

Unexpectedly, the application of individual droplets at the very high PA and PA-MPEG concentrations of did not lead to striking changes in the epidermal fine structure (Figure 6). Later observable epidermal changes were the consequences of the destruction of the mesophyll structure, and thus, the quick initial increase in transpiration was not causing lethal desiccant effects. This was also suggested by the fact that the still high use PA-MPEG concentration of about 30 g L^−1^ increased transpiration, but did not cause any phytotoxicity, even though this concentration resulted (see the calculation above) in a 5-fold higher dose per area than the cuticle mass (0.03–0.3 mg cm^−2^ for different species). Neither the increased efficacy with the used concentration nor the Hasten effect suggested a key role of transpiration. Although the transpiration effects of PA and PA-MPEG were measurable, they did not give a clear picture. For example, about 2 h after the adaxial application of PA-MPEG to amphistomatic pepper plants (experimentally preferred to *S. nigrum* due to lack of trichomes), adaxial transpiration rose from 20 (SD 4.6) mmol m^−2^ s^−1^ for untreated plants to 35 (SD 6.8) mmol m^−2^ s^−1^. In contrast, on the abaxial side with a higher stomatal number, transpiration rates decreased on average from about 66 mmol m^−2^ s^−1^ for the untreated plants to 44 mmol m^−2^ s^−1^, and the daily maximum transpiration rates were generally much higher with values of around 200 mmol m^−2^ s^−1^.

Cuticular transpiration was also measured with enzymatically isolated cuticles [56]. The addition of PA-MPEG to very dense common ivy cuticles increased transpiration by more than 10-fold. Cuticular transpiration is only minor contributing to total leaf water loss (a few percent with open stomata) of mature leaves. Not even significantly increased cuticular transpiration alone can explain phytotoxicity. However, for young expanding leaves or growing weeds, cuticular transpiration is the main source of water loss, and at least juvenile plant organs might be completely damaged.

Thus, we conclude that even when cuticular and/or stomatal transpiration increased, the observed wilting and desiccation symptoms were not caused by them. Instead, we suggest a combination of three factors that makes PA and PA-MPEG contact herbicides with desiccant action. First, high amounts of both herbicides are needed because the main targets are thylakoid membranes or chloroplast lipids. Plants can flexibly react to temperature or other stress factors and permanently repair membranes, having distinct membrane lipids and lipid metabolism with galactolipids and sulfolipids that directly come from photosynthetic products [57,58]. Disturbing this key plant function and large lipid compartment and the permanently running repair mechanism, requires high amounts, and a 30% load of the lipid. To cause such damage, PA and PA-MPEG need to reach that target. To do so, not only cuticular penetration, but also migration in the apoplast of cell walls and the xylem are required. Some alcohol ethoxylates (non-ionic surfactants) have been reported to increase transpiration at 0.5%, which cause phytotoxicity as necrotic tissues [43]. However, not even very high use concentrations (of these surfactants) such as that typical for PA or PA-MPEG, bring about a comparable desiccant effect such as that with PA and PA-MPEG. The putative reason is that such alcohol ethoxylates are not mobile in the mesophyll, and do not even allow locosystemic movement in treated leaves. In contrast, PA and PA-MPEG are probably more mobile, given their lower affinity to bio-membranes, but high amounts are still needed to disturb the thylakoid assembly. PA is a small anionic solute that is particularly mobile. We still do not know whether PA-MPEG is hydrolyzed after entering the epidermis or the mesophyll to form free PA, but with an octanol/water partition coefficient of a log *P* value of 2.5, it is already as nonmetabolized PA-MPEG a very mobile solute once it has penetrated the cuticle [59]. Yet still, high amounts of PA and PA-MPEG continue to be needed to disturb the thylakoid assembly.

Another aspect that we consider to be highly relevant, and even causal for desiccant action, is that as large amounts of PA or PA-MPEG enter the plant tissue, the bio-membranes in the chloroplast disintegrate and cause the release of lipids galactolipids and sulfolipids as well as FA from membrane lipids and/or PA to the cell walls and xylem and thus the total apoplast [11,57]. The surface tension of PA-MPEG is below 30 mN m^−1^, and both phospholipids and soaps have surface tensions of 35–40 mN m^−1^. This breaks the cohesion of water such that water supply for transpiration is reduced and causes wilting rather than increased transpiration rates at the cuticle or epidermal level. This could be the real cause of the observed desiccation effect. Further research is already underway to confirm these findings.

This should not be mixed up with recent observations showing that surfactants such as phospholipids in the xylem can contribute to stabilize water flow at negative pressure [60]. Schenk et al. [60] suggest that xylem surfactants have a high affinity to lipophilic areas in vessels, and while surfactants increase the probability and number of air bubbles, they can reduce embolism by their action to limit bubble size and ease re-bubble dissolution in xylem sap. In contrast, we suggest that the large amounts of surfactants resulting from either PA or PA-MPEG applications together with those released from membrane disintegration, potentially destroy this very stable mechanism of water uptake via the cohesion-tension. The site of actions is in the cell wall where the capillaries of cellulose fibers supply water that evaporates at the interface to the intercellular. Negative effects of surfactants on water transport by increased embolism have been shown to occur in the xylem, but not in the context of the MoA for killing weeds by affecting the capillary system in mesophyll cell [61]. With PA, water can no longer follow the steep gradient to the more negative water potential of unsaturated air. This could be a new target for other novel contact herbicide principles by interfering with the water cohesion-tension in the apparent free space of the cell wall.

## 4. Conclusions

Contact herbicides based on free PA have several disadvantages such as high use rates and water volumes, bad smell, and irritant factors, and they also require complex formulation. A novel ester, PA-MPEG, can reach and outperform the best PA benchmark, and is a ready-to-apply liquid with exceptional use properties. In contrast to free PA, the PA-MPEG is a significantly larger molecule that benefits from penetration enhancing adjuvants. An alkylated seed oil product (Hasten) increased cuticular permeability, directly giving better weed control. Even though performance was boosted with this adjuvant, the high application rates remained almost unchanged. Therefore, the product is preferred for precision application to specific sites, such as furrow application or with weed detection application systems, and drone application appears particularly interesting.

## Figures and Tables

**Table 1:** Test compounds, applied concentrations used and the pH of spray solutions with PA-MPEG.

Test Compounds	Description	Use Rate (% *v/v*) ^1^	pH Spray Mixture ^2^
Phosphoric acid	Solution–85 wt. % in H_2_O.	0.60	1.9
*D*-glucose	97.5% purity.	1.00	5.8
Potassium carbonate	99.0% purity.	1.00	10.3
*1*-decanol	99.0% purity.	1.00	6.1
Synergen^®^ TS 7 ^3^	Blend of docusate sodium and ethoxylated fatty alcohol (sum 100%).	0.15	5.8
Polyglykol 400 ^3^	Polyethylene glycol (PEG) with a molar weight of 400.	1.50	5.9
Genapol^®^ C 050 ^3^	Coconut fatty alcohol polyglycol ether with 5 EO.	1.00	5.8
Hasten^TM4^	Emulsifiable concentrate of esterified vegetable oil and non-ionic surfactants.	2.50	6.1

^1^ Rate based on label recommendation and previous trials. ^2^ Pelargonic acid ester of methyl polyethylene glycol (PA-MPEG) at 7.5% *v*/*v*. PA-MPEG pH: 5.8. ^3^ Clariant, (Muttenz, Switzerland). ^4^ Victorian Chemicals (Coolaroo, Australia).

**Table 2:** Phytotoxicity assessment.

Rate	Description
1	No damage
2	Slight symptoms (discoloration of tissue)
3	Slight necrotic spots
4	Strong symptoms (Complete necrosis)

**Table 3:** Impact of the test compounds on weed control (*Digitaria sanguinalis* and *Solanum nigrum*) 2 days after applications with 7.5% pelargonic acid ester of methylated polyethylene glycol (PA-MPEG) at 200 L ha^−1^ spray volume.

Test Compound	Concentration (%) ^1^	Weed Control (%)	
		*D. sanguinalis*	*S. nigrum*
None		29 d *	50 bc *
*1*-Decanol	1.00	43 a	74 a
Phosphoric acid	0.63	33 cd	59 b
D-Glucose	1.00	29 d	47 c
Potassium Carbonate	1.00	30 cd	48 c
Genapol C 050	1.00	31 cd	52 bc
Polyglycol 400	1.50	31 cd	53 bc
Synergen TS 7	0.15	36 bc	58 b
Hasten	2.50	39 ab	68 a

^1^ Concentration based on label recommendation and previous trials. * Means followed by common letters in a column are not significantly different by the Student–Newman–Keuls test at the 5% level of significance.

## Data Availability

Not applicable.

## Supplementary Materials

The following supporting information can be downloaded at: https://www.mdpi.com/article/10.3390/plants11030279/s1, Table S1. Weed control percentage (visual control ratings) of *Digitaria sanguinalis* and *Solanum nigrum*, 2 days after application of pelargonic acid ester methyl polyethylene glycol (PA-MPEG) at 7.5% alone and with tested compounds. Spray Volume 200 L ha^−1^; Table S2. Weed control percentage (visual control ratings) of *Digitaria sanguinalis*, 2 days after application of pelargonic acid ester methyl polyethylene glycol (PA-MPEG) at 7.5% with the addition into the spray tank of Hasten at different concentrations; Table S3. Weed control percentage (visual control ratings) of *Digitaria sanguinalis* and *Solanum nigrum,* 2 days after application of pelargonic acid ester methyl polyethylene glycol (PA-MPEG) at 7.5% alone and with tested compounds. Spray Volume 400 L ha^−1^.
