# Supplementary material for: Mechanistic Aspects and Effects of Selected Tank-Mix Partners on Herbicidal Activity of a Novel Fatty Acid Ester
Source: Plants (Basel). 2022 Jan 21;11(3):279. doi: 10.3390/plants11030279 (PMC8839195; doi:10.3390/plants11030279)
Supplement: Supplementary file 1 [file plants-11-00279-s001.zip › plants-1510304-supplementary.pdf]

SUPPLEMENTARY MATERIAL

Table S1: Weed control percentage (visual control ratings) of *Digitaria sanguinalis* and *Solanum nigrum,* 2 days after application of pelargonic acid ester methyl polyethylene glycol (PA-MPEG) at 7.5 % alone and with tested compounds. Spray Volume 200 L ha^{-1}.

Pest Code				*Digitaria sanguinalis*	*Solanum nigrum*
Days After Application (DAA)				2	2
Herbicide	Rate (%)	Test compound	Concentration (%)		
Untreated Check				0.00	0.00
PA-MPEG	7.50	None		28.50 ± 0.87 d	50.25 ± 3.45 bc
PA-MPEG	7.50	1-Decanol	1.00	42.50 ± 1.44 a	73.50 ± 3.01 a
PA-MPEG	7.50	Phosphoric acid	0.63	32.50 ± 0.87 cd	59.00 ± 3.46 b
PA-MPEG	7.50	D-Glucose	1.00	28.50 ± 0.87 d	46.75 ± 1.18 c
PA-MPEG	7.50	Potassium carbonate	1.00	30.25 ± 2.36 cd	47.75 ± 0.75 c
PA-MPEG	7.50	Genapol C 050	1.00	30.50 ± 1.66 cd	51.50 ± 2.22 bc
PA-MPEG	7.50	Polyglycol 400	1.50	30.50 ± 1.66 cd	52.50 ± 2.63 bc
PA-MPEG	7.50	Synergen TS 7	0.15	35.75 ± 0.48 bc	58.25 ± 1.97 b
PA-MPEG	7.50	Hasten	2.50	38.50 ± 0.87 ab	68.00 ± 3.11 a

Means followed by a common letter in a column are not significantly different by the Student–Newman–Keuls test at the 5 % level of significance.
Untreated check is not included in the analysis.

Table S2: Weed control percentage (visual control ratings) of *Digitaria sanguinalis,* 2 days after application of pelargonic acid ester methyl polyethylene glycol (PA-MPEG) at 7.5 % with the addition into the spray tank of Hasten at different concentrations.

Spray volume				200 L ha^{-1}	400 L ha^{-1}
Days After Application (DAA)				2	2
Herbicide	Rate	Adjuvant	Concentration (%)		
Untreated Check				0.00	0.00
PA-MPEG	7.50 %	None		27.60 ± 0.60 b	62.80 ± 0.80 d
PA-MPEG	7.50 %	Hasten	1.00	30.00 ± 1.84 b	69.40 ± 2.40 c
PA-MPEG	7.50 %	Hasten	2.00	35.40 ± 0.40 a	78.40 ± 2.93 b
PA-MPEG	7.50 %	Hasten	2.50	37.80 ± 0.97 a	85.20 ± 1.28 a
Beloukha	10.9 Kg a.i. ha^{-1}			34.40 ± 1.17 a	56.00 ± 1.00 e

Means followed by a common letter in a column are not significantly different by the Student–Newman–Keuls test at the 5 % level of significance.
Untreated check is not included in the analysis.

Table S3: Weed control percentage (visual control ratings) of *Digitaria sanguinalis* and *Solanum nigrum,* 2 days after application of pelargonic acid ester methyl polyethylene glycol (PA-MPEG) at 7.5 % alone and with tested compounds. Spray Volume 400 L ha^{-1}.

Pest Code				*Digitaria sanguinalis*	*Solanum nigrum*
Days After Application (DAA)				2	2
Herbicide	Rate (%)	Adjuvant	Concentration (%)		
Untreated Check				0.00	0.00
PA-MPEG	5.00	None		26.25 ± 1.25 e	35.75 ± 1.49 e
PA-MPEG	5.00	Hasten	2.50	31.25 ± 1.75 d	43.00 ± 2.71 d
PA-MPEG	6.00	None		31.25 ± 1.25 d	49.25 ± 2.17 c
PA-MPEG	6.00	Hasten	2.50	36.25 ± 1.25 a-d	55.00 ± 2.04 bc
PA-MPEG	7.00	None		33.25 ± 1.18 cd	55.50 ± 2.10 bc
PA-MPEG	7.00	Hasten	2.50	37.50 ± 1.44 abc	60.50 ± 1.66 ab
PA-MPEG	8.00	None		35.75 ± 1.49 bcd	57.75 ± 0.75 b
PA-MPEG	8.00	Hasten	2.50	40.00 ± 1.22 ab	67.00 ± 3.39 a
PA-MPEG	9.00	None		37.25 ± 1.03 abc	60.00 ± 1.22 ab
PA-MPEG	9.00	Hasten	2.50	40.75 ± 0.75 ab	66.25 ± 1.25 a
PA-MPEG	10.0	None		38.50 ± 0.87 abc	61.25 ± 1.75 ab
PA-MPEG	10.0	Hasten	2.50	41.50 ± 0.87 a	66.50 ± 0.50 a

Means followed by a common letter in a column are not significantly different by the Student–Newman–Keuls test at the 5 % level of significance.
Untreated check is not included in the analysis.
